# Radiosurgical Ablation of the Renal Nerve in a Porcine Model: A Minimally Invasive Therapeutic Approach to Treat Refractory Hypertension

**DOI:** 10.7759/cureus.1055

**Published:** 2017-02-26

**Authors:** Nikhilesh Bhatt, Sarah A Long, Edward A Gardner, Jonathan Tay, Elena Ladich, David Chamberlain, Thomas J. Fogarty, Patrick J Maguire

**Affiliations:** 1 Fogarty Institute for Innovation; 2 CyberHeart Inc.; 3 Acurray Inc.; 4 Cardiovascular Pathology, Memorial Regional Hospital; 5 Radiation Oncology, Reno CyberKnife; 6 Fogarty Insitute for Innovation

**Keywords:** renal nerve ablation, cyberheart, hypertension, cardiovascular diseases, radiosurgery, cardiac radiosurgery, cyberknife radiosurgery, minimally invasive

## Abstract

**Background:**

Hypertension is strongly associated with cardiovascular diseases such as heart failure, stroke, kidney disease, and has been correlated with an increased risk for heart attack. Current treatment regimens for hypertension are highly inadequate, with reports indicating that only 50.1% of the clinical population with the disease has their blood pressure under control.

**Objective:**

To study the feasibility of using minimally invasive radiosurgery to ablate the renal nerves as a novel treatment for refractory hypertension, and to assess the safety and efficacy of such an approach.

**Methods:**

A Hanford porcine (miniswine) model (N = 6) was used to investigate the feasibility of using the CyberHeart radiosurgical platform (CyberHeart Inc., Mountain View, CA, USA) to create safe renal nerve ablations. Norepinephrine (NE) levels were measured pre and post treatment. Additionally, renal nerve and arterial histology were studied to examine effect.

**Results:**

Plasma norepinephrine levels showed a decrease over the six-month time point. Urea, nitrogen, and creatinine levels showed no changes post procedure. Histology documented no significant arterial injury in targeted areas. Renal nerves documented histologic change consistent with nerve ablation.

**Conclusion:**

CyberHeart radiosurgery of the renal nerve is feasible and resulted in norepinephrine reduction and renal nerve injury consistent with radiosurgical targeted ablation.

## Introduction

Hypertension is strongly associated with cardiovascular diseases such as heart failure, stroke, kidney disease, and has been correlated with an increased risk for heart attack [1-2]. It has been reported that approximately 30% of the population over 18 years old has hypertension, and given the increasing prevalence of obesity, that number is anticipated to increase in the future [1-2]. Despite efforts that have improved awareness of the disease, hypertension treatment regimens for hypertension are highly inadequate. Studies have displayed that only 50.1% of the clinical population with hypertension has their blood pressure under control (defined as a level below 140/90 mmHg) [2-4].

The development of hypertension has been observed to be related to sympathetic nervous system activity [5-6]. More specifically, increased levels of sympathetic nervous system activity has been established to lead to high blood pressure [5]. Pharmacological treatments that target the sympathetic nervous system to treat hypertension have had limited success in treating hypertension [5]. In addition to the possibility of pharmacological treatment for hypertension to cause adverse side effects, a major concern with such treatment strategies is that they are ineffective for the large clinical population with refractory hypertension, which is defined as uncontrolled blood pressure despite the prescription of at least three hypertensive drugs [2, 5]. It is estimated that approximately 15% of the entire clinical population with hypertension (~76.4 million adults over 20 in the U.S.) are considered to have refractory hypertension [5-6]. Consequently, ablation of the renal sympathetic nerve has been an emerging treatment modality that is now being used to treat hypertension [5].

While the use of catheter ablation of the renal sympathetic nerve for the treatment of hypertension has been shown to lead to a decrease in blood pressure, the relatively new treatment approach has yet to produce consistently efficacious results [5, 7]. One proposed hypothesis for why catheter ablation has been observed to produce non-uniform results is because the procedure is done inside the artery, and as a result, the delivered energy has to be low in order not to damage the artery. This low energy may be insufficient to ablate the renal nerve, resulting in an ineffective procedure [7-8]. Additionally, it has been suggested that anatomic variations of nerve location with respect to artery location may also contribute to failure to ablate the renal nerve. Accordingly, there is a significant unmet clinical need for a new treatment modality for hypertension that produces uniformly efficacious results.

CyberHeart Inc. has pioneered the world’s first cardiac radiosurgery system (CRS) that can non-invasively ablate the heart with remarkable precision [9-10]. This innovative technology, which is anticipated to radically improve clinical outcomes for the millions of patients with cardiac arrhythmia, uses the CyberKnife (Accuray, Inc., Sunnyvale, CA, USA) radiosurgical platform to accurately transmit radiation to cardiac targets [9-10]. Given the exceptional degree of precision that the CyberHeart (CyberHeart Inc., Mountain View, CA, USA) CRS has displayed to date, it can be anticipated that the CyberHeart radiosurgery system (RS) has a promising potential to non-invasively ablate the renal nerve and provide a treatment for hypertension. The aim of this study was to evaluate the potential of the CyberHeart RS to provide a minimally invasive treatment for hypertension by examining the system’s ability to ablate the renal nerve in porcine models.

## Materials and methods

Six Hanford miniswine (Sinclair BioResources, LLC), weighing between 35 and 50 kg, were used for this study. The experimental protocol was approved by the Institutional Animal Care and Use Committee (IACUC) of the Sutter Memorial Research Institute, Sacramento, CA. All experimental procedures were performed in accord with the conditions described in the guide for care and use of laboratory animals of the National Institutes of Health (publication NIH 86-23, revised 2007) and under the Association for Assessment and Accreditation of Laboratory Animal Care (AAALAC) standard operating procedures (SOPs).

### Radiosurgical ablation procedure

**Figure 1:**
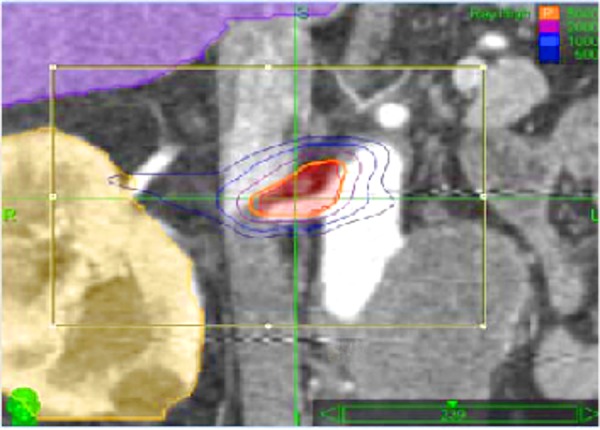
Multiplan™ Conformal Plan. MultiPlan™ contouring software interface for planning for targeting ablation of the aortic-renal ganglion and renal nerves circumferential to renal arteries. Red = target; yellow = kidney; green = pancreas; blue = spinal cord. Isodose Lines - Orange – 30 Gy; Purple – 20 Gy; Med. Blue – 10 Gy.

**Figure 2:**
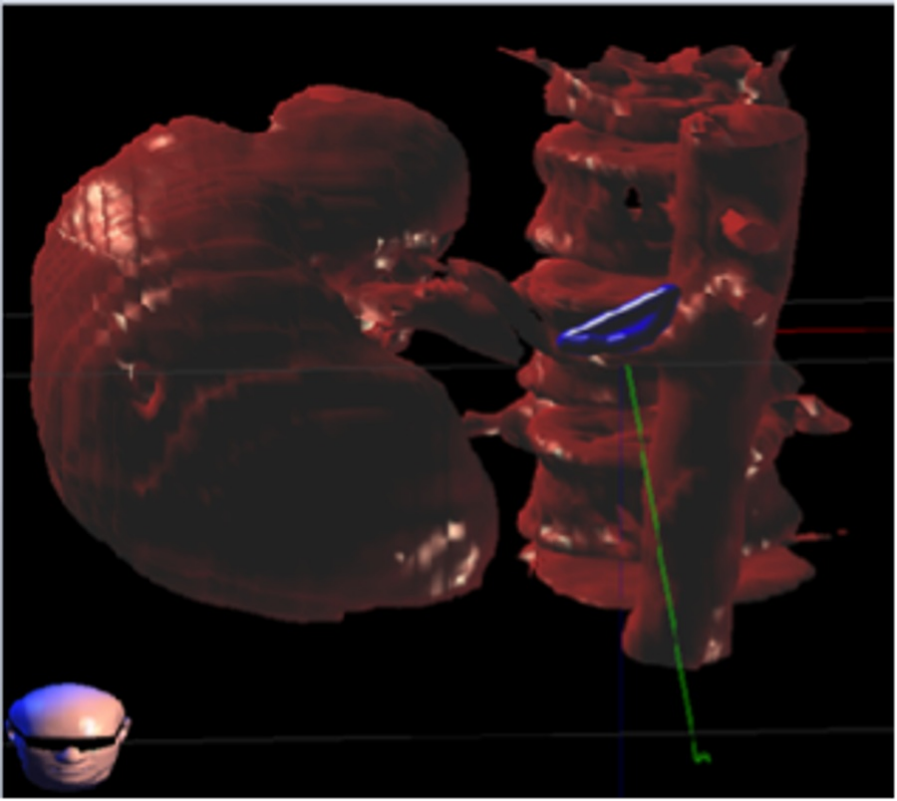
CardioPlan™ Targeting the Aortic-Renal Ganglion and Renal Nerves Circumferential to Renal Arteries.

Computed tomography (CT) scans were obtained and treatment planning was performed using CyberHeart’s CardioPlan™ software in combination with Accuray’s Multiplan™ for highly localized dose delivery for stereotactic radiosurgical ablation of the renal nerves using CyberKnife (Figures 1-2).

**Figure 3:**
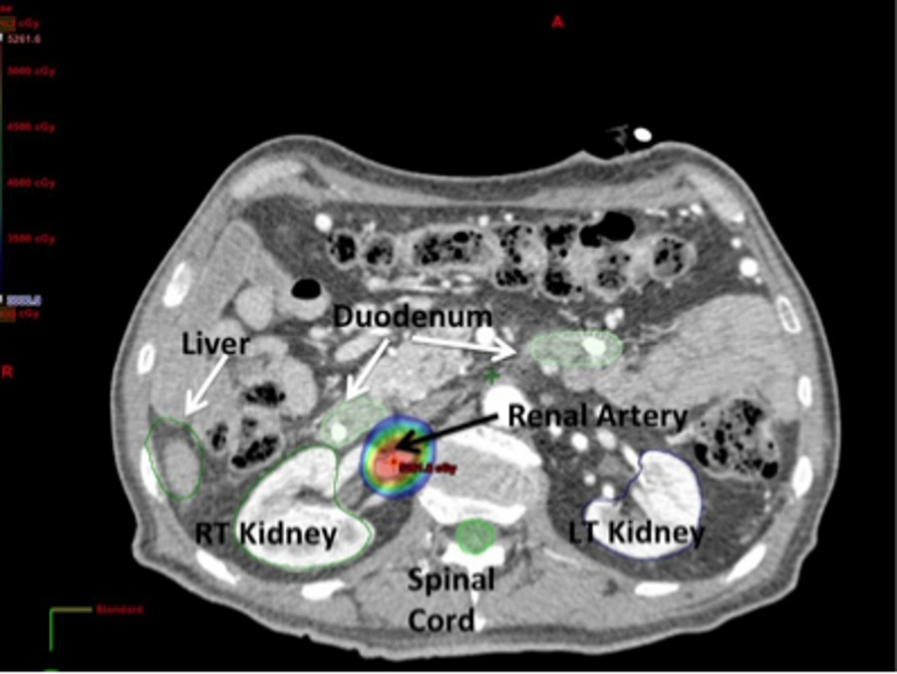
Axial CT Slice Renal Artery. A representative dose distribution of a prescription dose of 50 Gy to the renal artery. Through steep dose gradients, the dose to nearby organs is minimal. The colorwash shows a dose of [20 Gy] that does not reach the adjacent duodenum and renal hilum.

**Figure 4:**
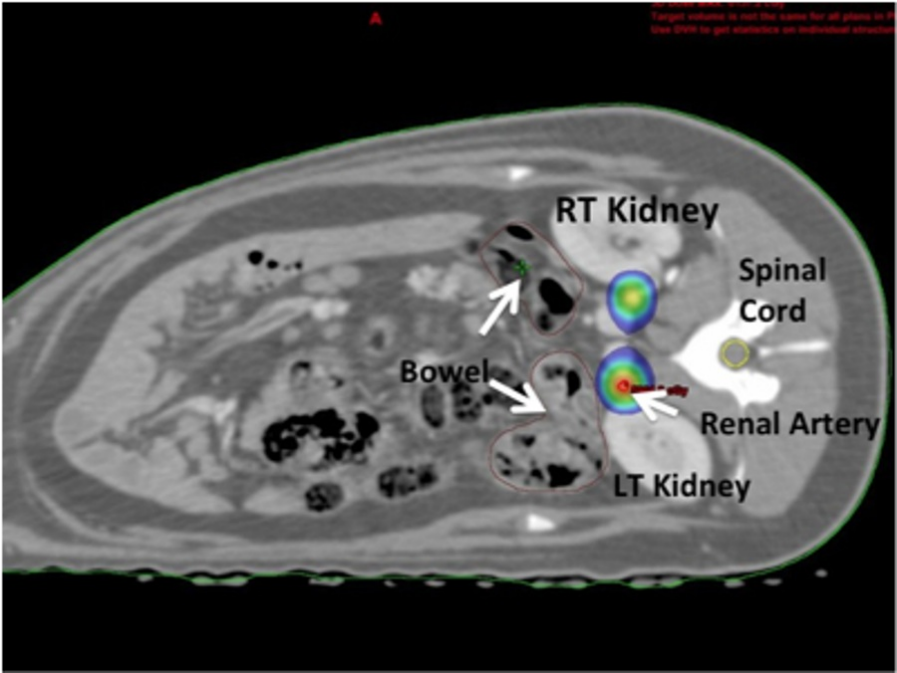
Axial CT Slice Renal Sympathetic Plexus. Axial CT slice depicting the dose distribution at and around the renal sympathetic plexus of a pig. Shown in colorwash is the 20 Gy isodose line that does not reach the adjacent kidneys, bowel, and spinal cord.

The miniswine underwent placement of a fiducial percutaneously under general anesthesia at the renal vein, through the inferior vena cava (IVC). In order to achieve renal nerve ablation, a prescription dose (40-50 Gy) of radiation was delivered under image guidance bilaterally to the aortic-renal ganglion or circumferential to the renal arteries using stereotactic radiosurgery (Figures 3-4). Synchrony motion management was used for intraprocedural tracking and the state-of-the-art CyberHeart RS was used to deliver radiation for the ablation.

### Longitudinal data collection

The animals were observed for six months with serum laboratories, urine analysis, and overall clinical and behavioral status. Norepinephrine (NE) levels were used as a surrogate for sympathetic denervation documented ablation. Measures of plasma and tissue NE levels (ng/ml) were recorded monthly through the follow-up period (4-6 months). At the end of this follow-up period, treated nerves, the aorta, renal vessels, and kidneys were studied histologically.

### Tissue preparation for histology

Following the protocol provided by CV Path Inc. (Gaithersberg, MD, USA), an en bloc segment of retroperitoneal tissue containing kidney, aorta, arteries, veins, vena cava, and ureter were removed following euthanasia. The kidney and associated arterial vasculature was pressure perfused using buffered formalin (10%). Arterial tissue was sectioned at intervals of 3-5 mm along the length of the artery, from ostium of the renal artery from the aorta to the hilum, in order to obtain cross sections. Sections were embedded in paraffin, mounted on slider and stained with hematoxylin and eosin for microscopic examination.

### Histopathologic evaluation and nerve assessment

The extent of structural and functional nerve injury was assessed by examination of histologic sections immunostained against neurofilament protein (NFP) for the recognition of axons within nerve fascicles in addition to tyrosine hydroxylase (TH), a functional indicator for norepinephrine synthesis. The intensity and distribution of staining was semi-quantified using a scoring system of 0 to 2; 0 = no reaction, 1+ = weak and/or patchy reaction to 2 + = strong reaction.

### Norepinephrine levels

At baseline and monthly, the animals received a sedative to allow for venipuncture.

### Tyrosine hydroxylase immunohistochemistry

Intensity of tyrosine hydroxylase is an indicator of efferent nerve activity and function. Tyrosine hydroxylase is a precursor to catecholamine synthesis that served to assess changes in renal sympathetic nerve function. A standard immunohistochemical staining protocol was used on tissue blocks from the sectioning of the renal artery and nerves. The intensity of the stain on the nerve tissue correlates with catecholamine synthesis by the sympathetic nerves. The semi-quantified scoring system used to approximate intensity of stain is as follows: 0-no reaction; 1-weak and/or patchy reaction; 2-strong reaction.

### Statistical analysis

Percentage of nerves per concentric arteria section are reported descriptively as mean +/- SD. Percentage reductions in renal artery norepinephrine levels of over time are expressed as a percentage of the baseline value.

### Histological analysis

CV Path Institute, Inc. (Gaithersburg, MD) performed all histologic analysis, and, accordingly, histology and immunohistochemistry (IHC) protocols were performed according to the CV Path standard operating procedures.

To assess long-term (six months) treatment reaction of the vessel, histology of the vessel, adjacent nerve fascicles, and adjacent organs (kidney, ureters, lymph nodes, and renal veins) were studied to assess treatment reaction. Ordinal data were collected for multiple parameters such as endothelial loss, arterial and venous medial injury (depth and circumference), inflammation, degenerative changes, and necrosis. These parameters were semi-quantified using a scoring system of 0 to 4: 0 = none; 1 = minimal, 2 = mild; 3 = moderate; and 4 = marked. In addition, distances from affected nerves to the media of the closest arterial segment were recorded.

## Results

### General health and physiological findings

All animals survived to the follow-up point. There were no in-life adverse events. Gross pathology was unremarkable. Norepinephrine levels decreased, on average, by 64% during follow-up. Histology documented moderate ablation changes in the renal nerves with lack of tyrosine hydroxylase staining. No renal artery demonstrated transmural medial injury or thrombus. Urea, nitrogen, and creatinine levels showed no changes post procedure.

### Radiosurgical ablation

Planning for radiosurgical ablation of the renal nerves using CardioPlan™ and CyberHeart radiosurgery (RS) was successful, with no adverse events occurring during the procedure.

### Histopathology and nerve assessment

With these modern treatment planning and delivery techniques, highly localized dose delivery with steep dose gradients was achieved. Morphometric analysis revealed that the mean distance of renal nerves from the lumen was 2.45 mm. The mean number of nerves examined was 150 per animal treated. 75% of the nerves exist between 1.0 and 3.0 mm from the lumen (Table 1). A lack of pathologic renal arteriopathy, and histologic changes in the aortic-renal ganglion target were observed.

**Table 1:** Average Injury Score for Six Pigs, Six Months After Delivery of 40 Gy to the Renal Artery. Mean Values of

Artery			Vein			Nerve
Endothelium	Media (depth)	Media (Circu)	Endothelium	Media (depth)	Media (Circu)	
0.63	0.85	0.65	0.29	0.24	0.10	1.69

### Tyrosine hydroxylase immunohistochemistry

Tyrosine hydroxylase staining was graded as described in the Methods and Materials section. Staining was generally found to decrease in intensity over time. The ablation observed with radiosurgery, consistent with scarring and fibrosis of tissue, is most evident at six weeks. The intensity of tyrosine hydroxylase staining was markedly reduced at six months.

### Safety observations

All animals survived to the follow-up point at six months. Treatment was successfully accomplished in all animals with no in-life adverse events or evidence of renal dysfunction, as indicated by stable serum creatinine levels over the follow-up period. No skin or behavioral changes were observed that might suggest animal discomfort.

### Norepinephrine levels representative of sympathetic nervous system activity

**Figure 5:**
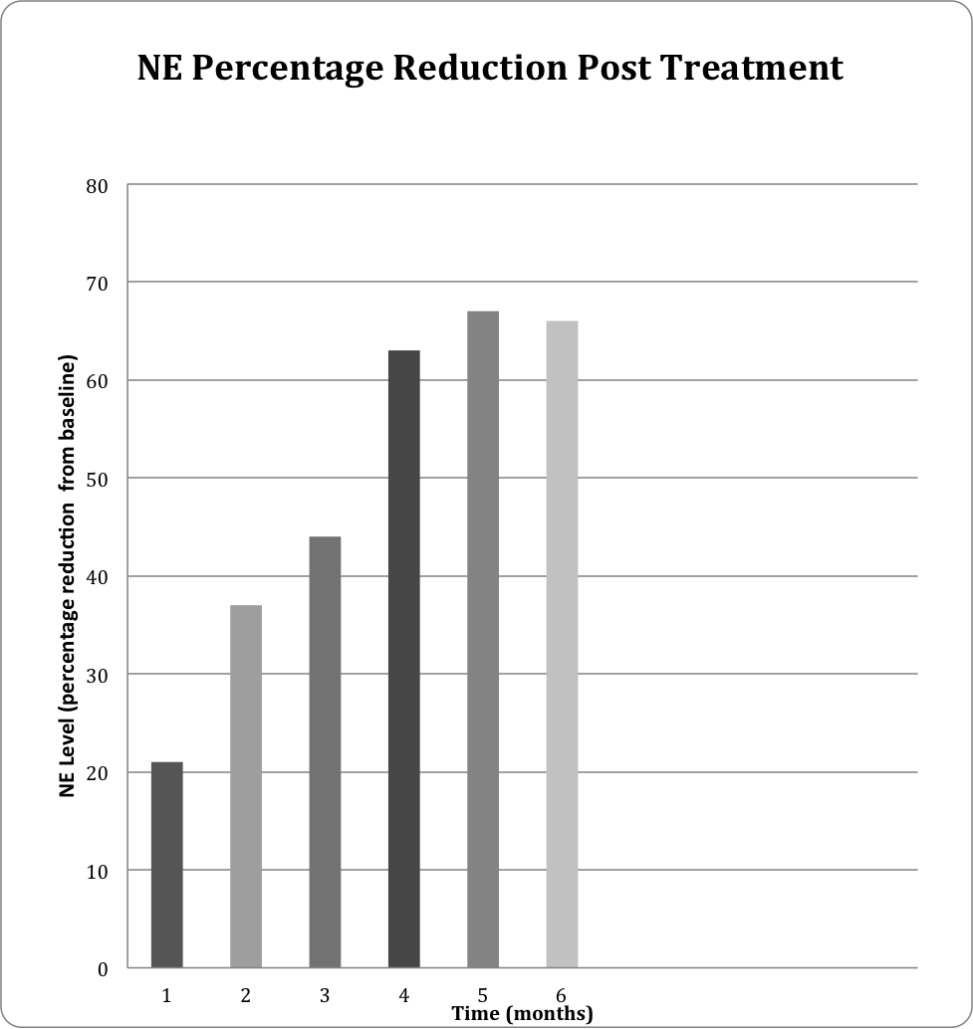
NE Reduction Post-CyberHeart RS Ablation of Renal Nerves. Time in Months (x-axis) Versus Percentage of NE Level Decrease from Baseline (y-axis).

Systemic norepinephrine levels are a common surrogate for sympathetic activity [11]. The average reduction observed in serum norepinephrine levels, on average, was 63%, with the median reduction at 73.5% during the follow-up period (Figure 5). As this is a radiosurgical feasibility study focused on the discovery of methodology, tracking, and targeting, blood pressure levels nor renal vein catecholamine levels were recorded.

### Histological analysis: renal nerves

**Figure 6:**
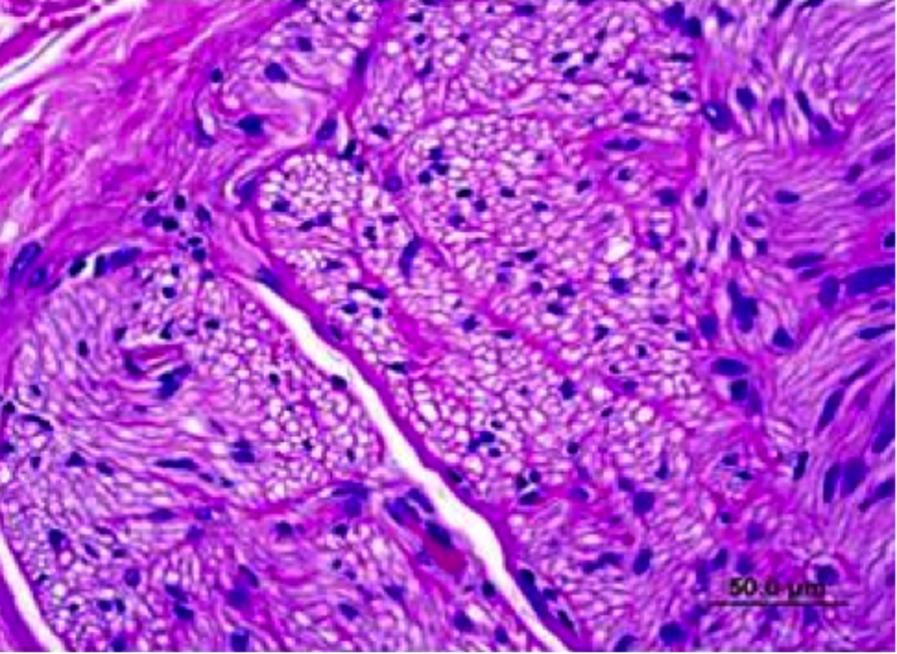
Six Month Nerve Fascicle with Pyknotic Nuclei and Vacuolization.

Gross pathology was unremarkable. Microscopic evaluation of the renal nerves showed evidence of damage to the nerves around treated renal arteries (Figure 6). Of the six animals treated at 40-50 Gy, changes were characterized as mild to moderate, with pyknotic nuclei and vacuolization.

**Figure 7:**
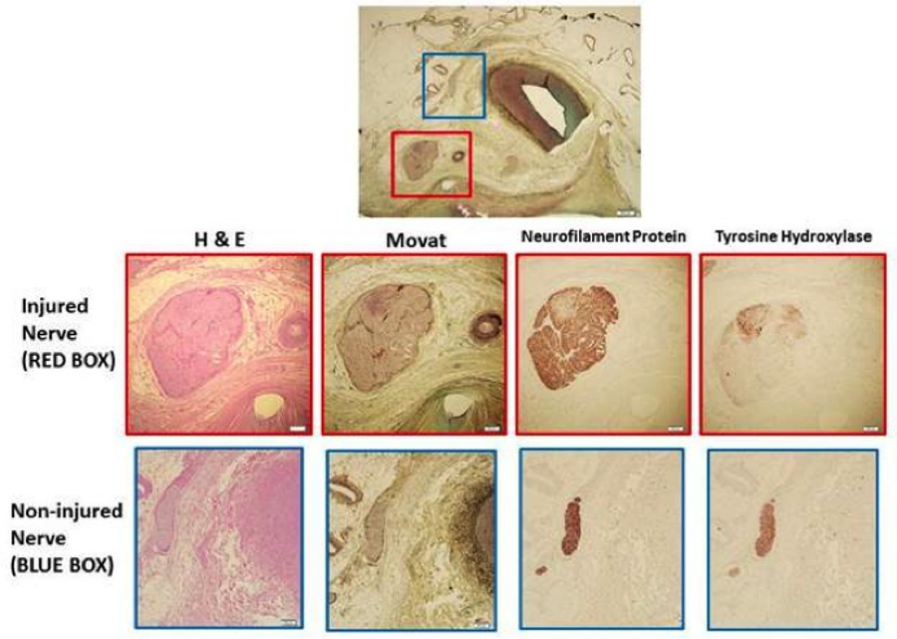
Six Month Immunohistochemistry Demonstrating Injured Nerve. Low power image shows right renal artery and surrounding perivascular tissue. Red boxes show an injured nerve with various stains. Both neurofilament protein and tyrosine hydroxylase show weak reaction. Blue boxes demonstrate non-injured nerves as a control.

Considerable attenuation in tyrosine hydroxylase immunoreactivity (THir) expression by immunohistochemistry is shown in Figure 7, with some vacuolar changes indicative of injury. This staining as well as immunostaining for neurofilament protein (NFP) show weak reaction in renal nerve at the end of the six month follow-up period (CyberHeart Inc., and CVPath Inc.). There was no histological or immunohistochemical evidence of damage to nearby spinal cord or spinal nerve root structures.

### Histological analysis: vessels

**Figure 8:**
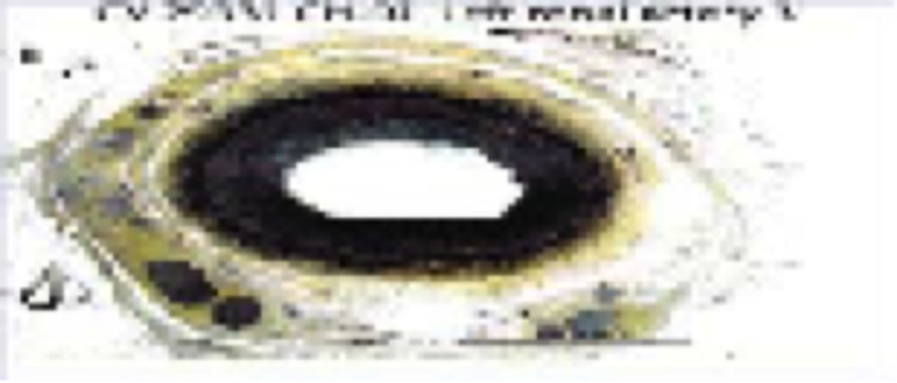
Six Month Renal Artery Vessel Wall After Radiosurgical Ablation. Vessel normal.

**Figure 9:**
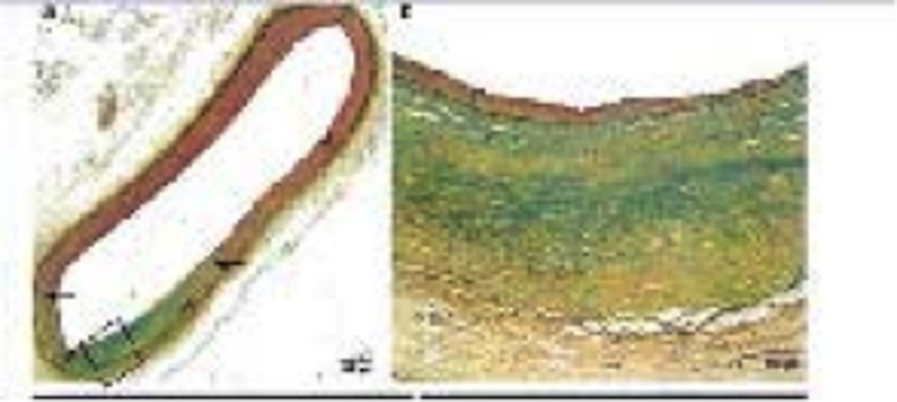
Six Month Renal Artery Vessel Wall After Radiofrequency Ablation. Wall abnormality present.

Histological analysis at the end of the follow-up period revealed no damage to the renal artery vessel wall in swine undergoing radiosurgical ablation via the technique described (Figure 8). Alternatively, ablation of the renal nerve using radiofrequency ablation caused injury to the vessel wall (Figure 9).

Each treated renal artery showed minimal to mild endothelial loss and medial changes. The endothelial loss scores vary from grade 0 to grade 3, but on average were minimal. Arterioles in treatment zones showed normal histologic architecture with minimal damage. No renal artery demonstrated transmural medial injury or thrombus.

## Discussion

The documented results demonstrate efficacy of the CyberHeart’s CardioPlan™ software in combination with CT scans and Multiplan™ in planning and executing the delivery of a localized, high dose of ionizing radiation to sympathetic renal nerves in a porcine model. The observed decrease in norepinephrine levels of radiosurgically ablated swine over the course of the study is evidence that supports CyberHeart RS successfully ablated renal nerves to decrease sympathetic nervous system activity. Moreover, because pathology indicated the renal nerves were successfully targeted bilaterally, it can be confidently suggested that radiosurgical ablation was effective in damaging the targeted nerves with a residual effect that lasted for at least six months.

Since the animals did not show any remarkable tissues other than the intended results, we conclude that this approach demonstrated safety in this preclinical model. Ionizing radiation maps provided additional evidence that critical nearby organs remained healthy and CyberHeart RS ablation of renal nerves was highly precise. Furthermore, histological analysis of renal arteries displayed that CyberHeart RS ablation of the renal nerves lead to improved safety of the surrounding arteries when compared to catheter-based radiofrequency ablations of the renal nerves.

While catheter ablation for the treatment of hypertension has shown the potential to possibly ameliorate the condition, its use for renal denervation has failed to display the capacity to consistently lower blood pressure. A recent randomized, sham-controlled, single blind, clinical trial (Symplicity HTN-3) rigorously investigated the efficacy of catheter ablation of the renal nerve for the treatment of resistant hypertension [12]. From the 535 patients with resistant hypertension that participated in Symplicity HTN-3, it was found that there was not a significant reduction in blood pressure when measured six months after the catheter ablation of the renal sympathetic nerve was completed when compared to the sham control [12]. Although it was suggested that additional clinical trials must still be performed to further elucidate the efficacy of catheter ablation of the renal nerve, it is important to note that this finding contradicted previous reports that indicated the treatment was effective at decreasing blood pressure [12]. One explanation for the findings from Symplicity HTN-3 that has been put forth is that the degree of renal nerve ablation achieved by each procedure (a measure not recorded from Symplicity HTN-3) was too low to produce a decrease in blood pressure [7]. The Symplicity HTN-1 clinical trial found that renal nerve ablation procedures are often incomplete; the average reduction of renal nerve spillover post-procedure was found to be only 47% [7-8]. Consequently, it has been suggested that catheter ablation of the sympathetic renal nerve is a deceptively complex procedure that often leads to non-uniform results because a surprisingly large percentage of the operations are incomplete [7].

While the predominant concern with the use of catheter ablation to treat hypertension is that it has failed to reliably decrease blood pressure, there are additional important concerns with the invasive procedure. Catheter ablation for the treatment of refractory hypertension fails to treat a significant cohort of the clinical population with hypertension, as the procedure has limited patient eligibility due to restrictions on glomerular filtration rate and inability to effectively operate on obscure renal artery anatomy [8, 13-18]. Another concern with using catheter ablation for renal denervation procedures is the possibility for intraoperative complications that can lead to life-altering outcomes. Complications of catheter ablation of the renal sympathetic nerve have been demonstrated to cause vascular lesions, local tissue damage to the area of operation, renal artery stenosis, thrombus formation, and edema [19-20]. Moreover, the invasive procedure has also been observed to result in coagulation necrosis of structures surrounding the targeted ablation tissue that were not intended to be ablated. When tissue that is not targeted is ablated, significant inflammation and injury can occur that results in the destruction of the subintimal architecture, including collagen and proteoglycans [21]. Furthermore, catheter ablation can cause unintended post coagulation necrosis that results in thinning of the arterial wall and potential weakness that compromises the ability of the arterial wall to withstand pressure [21].

It is crucial to realize that the mentioned complications that lead to life-altering outcomes or incomplete procedures that negligibly improve hypertension when using catheter ablation to treat the disease occur because the treatment is a deceitfully challenging, invasive procedure. With that important consideration in mind, the possibility of using radiosurgery, a minimally invasive treatment option that uses an external treatment approach, to ablate the renal nerves appears to be a logical treatment option that will circumvent the issues manifested by catheter ablation and will provide uniform, efficacious clinical outcomes to patients with hypertension. While stereotactic radiosurgery has primarily been used for oncologic applications historically, it has been convincingly demonstrated to be capable of precisely delivering single fraction ablation energy with sub-millimeter accuracy and has a well-documented ability to compensate for organ motion.

More recently, with the innovation of the cutting edge technology known as the CyberHeart RS, radiosurgery has been successfully applied to target cardiac targets. Currently, there are case reports that document the use of the CyberHeart RS to ablate the human heart. The case reports, which aimed to treat forms of cardiac arrhythmia, indicated that the CyberHeart RS has an incredible potential to accurately ablate the heart in a minimally invasive manner [22-23]. Previous preclinical studies have also provided evidence of the impeccable accuracy of the CyberHeart RS to ablate cardiac targets [24].

Considering the major limitations with the current standard of care for refractory hypertension and the demonstrated use of the CyberHeart RS to accurately ablate the heart, the results provided by this study have rather significant implications that indicate that the CyberHeart RS has a tremendous potential to provide a treatment for hypertension that provides improved clinical outcomes. CyberHeart RS avoids many of the challenges that occur with the invasive catheter ablation procedure because it is minimally invasive. Namely, concerns that occur with catheter ablation such as the ablation of non-targeted tissue and creating unintended vascular lesions simply do not exist with CyberHeart RS because the procedure utilizes a completely anatomic approach in which the attending physician uses the CardioPlan™ software to indicate exactly where the radiation should be delivered to cause an ablation before the procedure has even started. Moreover, the anatomic approach that CyberHeart RS allows removes concerns regarding intraoperative complication that arise during invasive procedures and is anticipated to lead to uniform clinical outcomes that have a much higher degree of renal sympathetic nerve ablation. Furthermore, it is highly likely that CyberHeart RS will significantly improve outcomes for individuals with abnormal renal artery vasculature and expand the treatable population of individuals with hypertension.

## Conclusions

This study aimed to use the CyberHeart radiosurgery system to ablate the renal sympathetic nerves in a large preclinical animal model by using an external, minimally invasive, radiosurgical approach. Treatment was successfully accomplished without any adverse events, and pathology indicated the renal nerves were accurately, bilaterally targeted. Norepinephrine levels, which were used as a surrogate to measure sympathetic denervation, had a significant decrease post-procedurally and provided further evidence that the radiosurgical ablation was successful.

Accordingly, this study has provided essential preclinical documentation that suggests CyberHeart radiosurgery has a tremendous potential to provide a novel therapy for the clinical population with hypertension that will improve patient outcomes. Future research is focused on investigating the use of the CyberHeart RS to treat hypertension in human subjects.
